# Status of underrepresented minority and female faculty at medical schools located within Historically Black Colleges and in Puerto Rico

**DOI:** 10.3402/meo.v21.29535

**Published:** 2016-03-09

**Authors:** Emily M. Mader, José E. Rodríguez, Kendall M. Campbell, Timothy Smilnak, Andrew W. Bazemore, Stephen Petterson, Christopher P. Morley

**Affiliations:** 1 Department of Family Medicine, SUNY Upstate Medical University, Syracuse, NY, USA; 2 Center for Underrepresented Minorities in Academic Medicine, Department of Family Medicine & Rural Health, College of Medicine, Florida State University, Tallahassee, FL, USA; 3 College of Medicine, SUNY Upstate Medical University, Syracuse, NY, USA; 4 Lerner Center for Public Health Promotion, Maxwell School of Citizenship & Public Affairs, Syracuse University, Syracuse, NY, USA; 5 Robert Graham Center, Washington, DC, USA; 6 Department of Family Medicine, University of Cincinnati, Cincinnati, OH, USA; 7 Department of Family Medicine, Georgetown University, Washington, DC, USA; 8 Department of Family Medicine, Virginia Commonwealth University, Virginia, USA; 9 Department of Health Policy, George Washington University School of Public Health, Washington, DC, USA; 10 Department of Psychiatry & Behavioral Sciences, SUNY Upstate Medical University, Syracuse, NY, USA; 11 Department of Public Health & Preventive Medicine, SUNY Upstate Medical University, Syracuse, NY, USA

**Keywords:** medical faculty, diversity, underrepresented minority, women, academic medicine

## Abstract

**Background and objectives:**

To assess the impact of medical school location in Historically Black Colleges and Universities (HBCU) and Puerto Rico (PR) on the proportion of underrepresented minorities in medicine (URMM) and women hired in faculty and leadership positions at academic medical institutions.

**Method:**

AAMC 2013 faculty roster data for allopathic medical schools were used to compare the racial/ethnic and gender composition of faculty and chair positions at medical schools located within HBCU and PR to that of other medical schools in the United States. Data were compared using independent sample *t*-tests.

**Results:**

Women were more highly represented in HBCU faculty (mean HBCU 43.5% vs. non-HBCU 36.5%, *p*=0.024) and chair (mean HBCU 30.1% vs. non-HBCU 15.6%, *p*=0.005) positions and in PR chair positions (mean PR 38.23% vs. non-PR 15.38%, *p*=0.016) compared with other allopathic institutions. HBCU were associated with increased African American representation in faculty (mean HBCU 59.5% vs. non-HBCU 2.6%, *p*=0.011) and chair (mean HBCU 73.1% vs. non-HBCU 2.2%, *p*≤0.001) positions. PR designation was associated with increased faculty (mean PR 75.40% vs. non-PR 3.72%, *p*≤0.001) and chair (mean PR 75.00% vs. non-PR 3.54%, *p*≤0.001) positions filled by Latinos/Hispanics.

**Conclusions:**

Women and African Americans are better represented in faculty and leadership positions at HBCU, and women and Latino/Hispanics at PR medical schools, than they are at allopathic peer institutions.

The low number of underrepresented minorities in medicine (URMM) is a persistent problem in both the general physician workforce and academic medicine. URMM faculty members are part of one or more of the following racial and/or ethnic groups: black, Latino/Hispanic, or Native American/Alaska Native. The face of health care has had an increasingly difficult time mirroring the face of our communities, and the implications for underrepresentation have been discussed in clinical (1, 2) and academic (3–5) arenas. Recent studies have concluded that non-white physicians tend to care for minority patient groups, and increasing the racial and ethnic diversity of the physician workforce, including medical faculty, may help eliminate health disparities (1, 6, 7). Furthermore, URMM and women faculty members are thought to play an important role in medical education by establishing mutually beneficial relationships with URMM and women medical students (8, 9). However, current numbers of URMM faculty are at disproportionately low levels compared with the national percentages of minorities in the general population (10–12), and the representation of women in academic medicine has failed to reach parity with men (13). Lack of mentorship (14–16), disproportionate remuneration (17), low institutional expectations of success (18), as well as overt or covert racial, ethnic, and gender discrimination (14, 15, 19–22) have been implicated in fueling disparities in medical faculty composition.

Historically, dating back to the turn of the last century, one avenue for potential URMM faculty to enter academia was through Historically Black Colleges and Universities (HBCU). However, the Flexner Report of 1910, which was intended to standardize medical education by creating higher standards for educational training as well as admission to medical school (23), resulted in the closing of several medical schools, five of which were located at HBCU. In fact, before the Flexner Report, there were seven medical schools located at HBCU, and afterward there were only two, both of which were private institutions (23). Recent meetings organized under the *Beyond Flexner* project of George Washington University (24) concluded that the Flexner Report has had long-standing consequences on the nation's medical workforce, including the proportion of graduates from minority populations, and that increased diversity of the medical workforce is a means to excellence in education, practice, and health equity.

In addition to the changes developed from the Flexner Report, the dismantling of affirmative action has impacted the representation of URMM and women in academic medicine. Affirmative action was created to ‘level the playing field’ (25) for underrepresented groups in the workplace and academic institutions, and may have facilitated the ability of the institution of academic medicine to ensure that certain groups were represented as part of the whole and led to an increased presence of URMM (specifically black) and women faculty in U.S. medical institutions. However, with the continued dismantling of affirmative action, growth in the percentage of URMM academic physicians has flattened since 1994 (26, 27). Currently, URMM make up 8% of all medical faculty positions, which is only a 1% increase from their level of representation from over 20 years ago in 1993 (10, 12).

Attempts to further define the importance of increasing the representation of URMM and women faculty must be grounded in an understanding of current levels of diversity in academic medicine. To achieve this end, we compared the racial/ethnic and gender faculty composition at HBCU and Puerto Rican (PR) medical schools with that at their allopathic peer institutions in hopes of identifying if differences in faculty composition exist across institutions. The development of PR medical schools was uniquely shaped by the geographic and historical relationship between Puerto Rico and the United States, and these institutions continue to play an important role in the training of Latino/Hispanic health professionals. Since Latinos/Hispanics represent the largest ethnic or racial minority in the United States (28), PR medical schools were included in our analysis to account for their contribution to diversity in academic medicine.

## Methods

In this cross-sectional evaluation, we sought to test the hypotheses that HBCU and PR medical schools have (1) higher female and (2) higher URMM faculty ratios when compared with non-HBCU, non-PR allopathic U.S. medical schools, utilizing basic descriptive statistics and bivariate comparisons. This research was granted an exemption from review by the Institutional Review Board of SUNY Upstate Medical University (FWA #00005967, IRB Registration #00000391).

### Variables

The ratio of medical school faculty and chairs by race, ethnicity, and gender at U.S. allopathic medical schools was calculated from 2013 Association of American Medical Colleges (AAMC) faculty rosters (29). The rate of employment for each racial (African American, Native American/Alaska Native, Asian, Native Hawaiian/Other Pacific Islander, white), ethnic (Latino/Hispanic), and gender (Female/Male) category was calculated by taking the number of faculty/chairs reported for each category over the total number of faculty/chairs reported for each institution. Dummy variables representing HBCU (1/0) medical school designation and medical school location in Puerto Rico (1/0) were each created using publically available information for each institution.

### Analysis

Following the calculation of basic descriptive data, the mean percentage of faculty and chair positions filled by URMM racial/ethnic groups and women was compared between HBCU and non-HBCU medical schools, and between medical schools located within and outside Puerto Rico using independent sample *t*-tests. The impact of additional institutional characteristics (e.g., public/private designation and a proxy indicator of institutional commitment to social justice (30)) on these associations was further evaluated through ordinary least squares (OLS) linear regression. Statistical significance was determined at the 0.05 level. All analyses were conducted using SPSS Statistics Version 22 (IBM, Armonk, NY).

## Results

Data on faculty composition were available from 129 U.S. allopathic medical schools; the majority of these schools were public (68%). Data on chair composition were available from 126 U.S. allopathic medical schools, of which 63% were public. Three of the medical schools included in analysis were designated as HBCU, and three separate medical schools within the sample were located in Puerto Rico. All HBCU in the sample were private schools, and two of the three PR schools were private.

The percentage of faculty and chair positions filled by women was, on average, higher than the percentage of URMM hired in these positions at U.S. medical schools. However, the range of percent medical school faculty and chair positions filled by African Americans and Latinos/Hispanics was much wider than that of women. Summary statistics of the racial/ethnic and sex composition of medical school faculty and chair positions can be found in Table 1.

**Table 1:** Summary statistics of racial/ethnic and sex composition of faculty and chair positions at U.S. Allopathic Medical Schools, 2013

	Mean (SD)	Minimum	Maximum
Medical school faculty composition (*N*=129)			
% Female	36.62 (5.35)	25.73	51.34
% African American	3.92 (8.86)	0.00	71.30
% Asian	12.42 (5.77)	0.94	34.94
% Native American	0.16 (0.29)	0.00	2.50
% Hawaiian-OPI	0.13 (0.39)	0.00	3.01
% Hispanic/Latino^a^	5.43 (12.73)	0.00	92.31
% White	58.76 (14.48)	0.94	86.10
Medical school chair composition (*N*=126)			
% Female	15.93 (90.4)	0.00	50.00
% African American	3.88 (11.33)	0.00	80.00
% Asian	5.36 (5.80)	0.00	37.50
% Native American^b^	N/A	N/A	N/A
% Hawaiian-OPI	0.05 (0.56)	0.00	6.25
% Hispanic/Latino	5.24 (14.22)	0.00	95.45
% White	80.81 (19.78)	0.00	100.00

a *N*=126.

b No chair positions were filled by Native American/Alaska Native individuals in 2013.

Comparisons of the mean percentage of medical school faculty positions filled by women and URMM indicate that HBCU had a significantly higher average percentage of faculty positions filled by both women and African Americans (*p*=0.024 and *p*=0.011, respectively); this pattern was repeated when comparing the mean percentage of chair positions in academic medicine between HBCU and non-HBCU (*p*=0.005 and *p*≤0.001, respectively). PR medical schools employed a significantly higher average percentage of Latino/Hispanics in both faculty and chair positions compared with schools outside Puerto Rico (*p*≤0.001) and also employed a significantly higher average of women in chair positions (*p*=0.016). Results of the mean comparisons can be found in Table 2.

**Table 2:** Mean percentages of U.S. Allopathic Medical School faculty and chair composition, by Historically Black College and University Designation and Puerto Rico Location, 2013

	HBCU	Non-HBCU	*p*^a^	PR medical school	Non-PR medical school	*p*^a^
Medical school faculty composition						
% Female	43.5	36.5	0.024	42.85	36.48	NS
% African American	59.5	2.6	0.011	0.00	4.01	≤0.001
% Asian	11.9	12.4	NS	3.17	12.64	0.019
% Native American	0.419	0.154	NS	0.00	0.16	≤0.001
% Hawaiian-OPI	0.0	0.14	NS	0.00	0.14	≤0.001
% Hispanic/Latino^b^	3.3	5.5	NS	75.40	3.72	≤0.001
% White	15.23	59.79	0.004	23.68	59.60	NS
Medical school chair composition						
% Female	30.1	15.6	0.005	38.23	15.38	0.016
% African American	73.1	2.2	≤0.001	0.00	3.98	≤0.001
% Asian	3.3	5.4	NS	0.00	5.50	≤0.001
% Native American^c^	N/A	N/A	N/A	N/A	N/A	N/A
% Hawaiian-OPI	0.0	0.1	NS	0.00	0.05	NS
% Hispanic/Latino	2.6	5.3	NS	75.00	3.54	≤0.001
% White	13.33	81.19	0.007	5.96	81.37	0.001

a Independent sample *t*-test.

b HBCU (*N*=3), Non-HBCU (*N*=123).

c No chair positions were filled by Native American/Alaska Native individuals in 2013.

Additional modeling through OLS regression did not reveal any statistically significant relationships between the faculty and chair composition ratios and public/private status or a proxy indicator of institutional commitment to social justice (data not shown).

## Discussion

Increasing the proportion of URMM and women in faculty and chair positions in U.S. academic medicine requires assessment of current performance in these areas across the various types of medical schools. The results of these analyses indicate that HBCU and PR medical schools are greatly outperforming other medical schools in the employment of women and URMM in faculty and leadership positions.

Across all institutions, women were, on average, hired in a larger percent of faculty and chair positions compared with URMM. It is possible that overcoming the underrepresentation of women in faculty positions at academic medical institutions has been less challenging compared with racial/ethnic diversification due to the increased volume of eligible female candidates for these positions. Between 1980 and 2011, the number of women graduating from medical school increased from 3,524 to 8,396, reaching near parity with men graduates (8,896) (31). The approximate parity observed between men and women medical school graduates is not, however, mirrored in employment in academic medicine (13). Our results show that HBCU and PR medical schools are associated with increased hiring of women in faculty and leadership positions when compared with other medical schools. Particularly, both institutions were associated with a higher percentage of women working in leadership positions as department chairs. These findings indicate that women are experiencing increased career mobility at HBCU and PR academic medical institutions compared with those employed in the general population of allopathic medical schools. Lending support to this conclusion, current assessments by others indicate that the majority of women faculty in academic medical institutions, generally, are employed at lower academic ranks (e.g., instructor and assistant professor) (32).

Despite the modest successes of targeted efforts to increase the representation of minority groups in the health professions – such as the AAMC's education pipeline program Project 3000 by 2000 (33) – the level of progress seen among women in academic medicine has not been mirrored in the number of URMM graduates from U.S. medical schools, and URMM continue to represent only a small fraction of medical school graduates. In 2011, of all medical school graduates, 6.6% were African American, 7.8% were Latino/Hispanic, 0.8% were Native American/Alaska Native, and 0.3% were Native Hawaiian/Other Pacific Islander (31).

It is important to note that the increased diversity of academic faculty and chair positions at HBCU and PR medical schools observed in this study's results is not inclusive of all URMM racial/ethnic groups; rather, HBCU and PR medical schools hire increased proportions of specific minority groups. HBCU medical schools have a larger proportion of African Americans filling faculty and chair positions compared with other allopathic institutions, but are not associated with increased employment of other URMM groups in these positions, such as Native American/Alaska Natives or Latinos/Hispanics. Similarly, PR medical schools employ a larger proportion of Latinos/Hispanics holding faculty and chair positions compared with other allopathic institutions, but are not associated with increased employment of other URMM racial/ethnic groups. After considering the geographic and historical contexts of both HBCU and PR medical schools, these observations are not surprising. In fact, the institutions responsible for graduating the largest numbers of URMM physicians also hire larger proportions of those same URMM groups in faculty and leadership positions. From 1980 to 2012, Howard University and Meharry Medical College (both HBCU) were responsible for graduating the most black/African American physicians in the United States. Additionally, the three U.S. medical schools graduating the most Latino/Hispanic physicians in the same time period were Hispanic-serving institutions in Puerto Rico (34).

The ability of medical schools to hire URMM and women in faculty and chair positions is dependent upon the supply of qualified individuals to fill those positions; thus, the lack of diversity in faculty and leadership positions in U.S. medical schools may partly reflect the lack of diversity in medical school graduates and faculty development candidates. This relationship does not, however, fully explain the consistently low levels of diversity in medical faculty and chair positions across U.S. medical schools. The representation of URMM and women in faculty and chair positions in academic medicine remains disproportionately low compared with the representation of these groups both in the general U.S. population and among medical school graduates.

While the results presented above do not tell us why HBCU and PR medical schools do better at hiring women and individuals from specific URMM groups, explicit practices at institutions such as HBCU or PR medical schools that favor the recruitment of URMM and women faculty members may only be part of the equation. It is equally probable that faculty from URMM groups and women are more likely to seek employment at such institutions. Similarly, the promotion or recruitment of department chairs from among URMM and women faculty may reflect explicit institutional practices or may be a reflection of the higher percentage of URMM and women faculty graduating from or employed at these institutions.

The fact that the observations made within this study are not surprising is itself an area of concern. The results of this study indicate that racial and ethnic silos persist in medical education in the United States. The expectation that HBCU medical schools and PR medical schools will hire and promote the individuals of the racial and ethnic groups historically targeted by these institutions is a reflection of the continued de facto separation of these groups from the mainstream medical education system. Efforts to increase the diversity of the medical student body, faculty, and leadership will be enhanced by an increased understanding of the mechanisms that perpetuate the lack of diversity, as well as those that promote the inclusion of diverse groups at all levels of medical education across U.S. academic medical institutions.

### Limitations

The data on race were obtained from the AAMC faculty roster, which was downloaded at the Florida State University College of Medicine in 2014. The data used for this study were taken from the 2013 roster, as it was the most recent data available at the time of analysis. The data have three main limitations: the use of the term ‘African American’, the separation of ethnicity from race, and the limited availability of institutional data. African American is used as a surrogate for black race, but it is not limited to black race. Africans of other races (most prominently Asian and white) can be included in that group and are included in some institutions. We suspect, however, that this is a very small number with a negligible effect on our findings.

In reading the results of this study, it is also important to take into account how ‘black’ is socially constructed and self-identified in Puerto Rico. Historically, on the island of Puerto Rico, one was considered white if one had a ‘legally white’ parent in the last four generations. For this reason, the majority of Puerto Ricans were considered white and self-identified as such (35). At the same time, anyone who has one black ancestor has been historically considered black on the U.S. mainland (36). Thus, many Puerto Ricans discover different racial identification customs when they immigrate to the U.S. mainland. Additionally, other racial categories frequently used in Puerto Rico do not easily correspond to the black/white categorization used in the U.S. mainland (37). Due to these contextual factors related to self-identification, we suspect that the number of black faculty employed in PR medical schools, by U.S. mainland measures, is likely higher than what is reported.

Another limitation stems from the separation of the race and ethnicity data sets. The merged data set we are currently using has Latinos/Hispanics listed discretely as white, black, Asian or Native American/Alaska Native. Evidence from the 2010 U.S. Census suggests that 97% of respondents in the United States consider themselves of only one race (38). If that is the case, it would seem that Latinos/Hispanics of mixed race would declare their race in a similar pattern to the general U.S. population. We suspect Latino/Hispanic ethnicity is not a confounder in our current study, but further study using the ethnicity data from the AAMC can likely clarify this issue.

There are a myriad of factors that may influence gender and URMM recruitment and advancement in the field of medical education beyond the fact that an institution is either historically black or located in Puerto Rico. However, due to the limited nature of the data to which we had access, we were unable to investigate the impact of other important institutional factors on faculty and chair diversity, including student body composition, and the age distribution of faculty and chairs by race, ethnicity, and gender. The analysis of additional data will be required to determine what specific institutional factors influence the level of diversity in academic medical faculty and leadership positions.

We were also unable to evaluate the representation of women URMM in leadership positions in medical education in our analyses due to limitations in the available data. While our results show that HBCU and PR medical schools hire a larger proportion of women in faculty and chair positions, we were unable to investigate the representation of minority women in these positions at HBCU and PR medical schools. Additional data must be collected and analyzed to determine if HBCU and PR medical schools hire and retain more women who are of URMM racial and ethnic groups in faculty and chair positions.

## Conclusions

If we are to increase the diversity of faculty and leadership in academic medicine, as well as the diversity of the general physician workforce in the United States, we may need to explicitly broaden the pool of recruitment and matriculation into medical school. Cultural and historical differences aside, these findings suggests that HBCU and PR medical schools may be implementing policies regarding recruitment and hiring practices, as well as institutional support structures, which promote gender and racial/ethnic diversity in faculty and leadership positions. Future research seeking an in-depth understanding of these policies may yield valuable insight into strategies to promote diversity across academic medical institutions.

## Presentations

This project was accepted for presentation at the 2015 Society of Teachers of Family Medicine Conference on Medical Student Education and has been accepted for presentation at the 2015 Society of Teachers of Family Medicine Annual Conference.
